# Association of serum and hair antioxidant minerals with an oxidative stress marker in relation with characteristics of healthy adults: a cross-sectional study

**DOI:** 10.1038/s41598-024-53725-6

**Published:** 2024-02-08

**Authors:** Yoo-Ree Kang, Mi-Kyung Sung, Hyun-Wook Baik, Mi-Kyeong Choi

**Affiliations:** 1 Department of Food and Nutrition, Sookmyung Women’s University, Seoul, 04310 Republic of Korea; 2 Department of Health Promotion and Internal Medicine, National Center for Mental Health, Seoul, 04933 Republic of Korea; 3 Department of Food and Nutrition, Kongju National University, 54 Daehak-ro, Yesan, 32439 Republic of Korea

**Keywords:** Oxidative stress, Antioxidants, Trace elements, 8-hydroxy-2′-deoxyguanosine (8-OHdG), Zinc, Chromium, Biochemistry, Health care

## Abstract

Excess oxidative stress generated in the body causes various types of cellular damage, including DNA damage. Certain trace minerals act as antioxidants by functioning as cofactors for antioxidant enzymes. This study was conducted to evaluate the serum and hair concentrations of major antioxidant trace minerals (zinc, manganese, selenium, and chromium) and to determine the association between the oxidative stress marker urinary 8-hydroxy-2′-deoxyguanosine (8-OHdG) and serum or hair antioxidant trace mineral concentrations, according to the general characteristics of healthy adults. Study participants were selected after screening, and 108 participants aged 19–69 years were finally included. Serum and hair trace mineral concentrations were analyzed using inductively coupled plasma mass spectrometry, and urine 8-OHdG levels were quantified using an ELISA kit. Results showed that urinary 8-OHdG levels were significantly higher in exercisers than in those who did not exercise. Correlation analysis revealed that urinary 8-OHdG was negatively correlated with hair zinc in participants over 60 years of age and with poor health status, and positively correlated with hair chromium in participants with irregular dietary habits. In conclusion, these results suggest that urinary 8-OHdG is particularly correlated with hair zinc and chromium levels. Additional large-scale epidemiological studies are needed to generally confirm these findings.

## Introduction

Oxidative stress is an imbalance between the production of reactive oxygen species (ROS) and the biological ability to detoxify reactive intermediates or repair damage caused by ROS^1^. ROS are generated during redox reactions in the body and are produced by both exogenous factors, such as smoking and alcohol consumption, and endogenous factors, such as oxidative phosphorylation and redox enzyme activity in the mitochondria^2^. The main nutrients affected by oxidative stress in the body are fat and proteins, and DNA is also sensitive to oxidative stress. Excessive ROS levels have been implicated in the development of cancer, diabetes, inflammatory diseases, ischemia and reperfusion injury, neurodegenerative disorders, and aging^3^. In particular, DNA damage may lead to potentially carcinogenic mutations^4^. 8-Hydroxy-2-deoxyguanosine (8-OHdG) is one of the major forms of oxidative lesions caused by free radicals and has been widely used as a primary marker to measure the extent of DNA oxidative damage and carcinogenesis because it is a relatively accurate indicator of oxidative damage and repair activity in DNA^5^. 

When the body is exposed to oxidative stress, it triggers mechanisms to counter it through enzymatic and non-enzymatic defenses^6^. Enzymatic defense mechanisms directly affect ROS production and involve enzymes such as superoxide dismutase (SOD), catalase (CAT), and glutathione peroxidase (GPX). Non-enzymatic defense factors might be endogenous (those that eukaryotic cells may synthesize) or exogenous (those that must be obtained through diet). Endogenous factors include glutathione (GSH), α-lipoic acid, melatonin, coenzyme Q10, and ubiquinone, which are responsible for scavenging several ROS and supporting the activities of other antioxidants (vitamins C and E). Exogenous factors include vitamins C and E, carotenoids, polyphenols, flavonoids, organosulfur, and epigallocatechin-3-gallate. They reduce oxidative stress by inhibiting the activity of free radicals. In particular, vitamin C is an effective scavenger of ROS that directly and indirectly activates oxidized vitamin E, GSH, and carotenoids^7^.

For the proper functioning of defense mechanisms against oxidative stress, the presence of antioxidant trace minerals that directly and indirectly affect enzymatic and non-enzymatic defense mechanisms is essential. Zinc acts in the form of Cu/Zn-SOD, where it is bound to SOD with copper and has an indirect effect on the mechanism of action of CAT, serving as an activator of the mechanism^8^. Manganese acts in the form of MnSOD, which is the most important enzyme for removing ROS from mitochondria^9^. Selenium is an essential dietary element that plays a central role in maintaining good health by producing approximately 25 different selenoproteins, which are part of the active site of the antioxidant enzyme GPX^10^. GPX is a selenium-dependent redox enzyme that uses H_2_O_2_ or an organic hydroperoxide as an oxidant and the tripeptide GSH as an electron donor to remove ROS from the body^11^. Chromium can be broadly categorized into hexavalent and trivalent forms. Trivalent chromium plays a role in glucose regulation in type 2 diabetes and is involved in lipid and protein metabolism^12^. In particular, chromium appears to function as an antioxidant enzyme cofactor in patients with diabetes, preventing the amount and activity of the antioxidant enzymes Cu/ZnSOD and GPX from being reduced as a result of the disease; however, the exact mechanism for the enzymatic cofactor remains unclear^13,14^.

As our understanding of the functions of antioxidant trace minerals and analytical techniques improve, the number of studies on the roles of antioxidant trace minerals in disease treatment and prevention is expected to increase. Therefore, to facilitate relevant studies, it is important to determine the normal ranges of trace mineral concentrations of antioxidants in each population. Antioxidant trace mineral concentrations in the body are quantified by analyzing serum, hair, and urine. Comparative studies of trace mineral concentrations using both serum and hair in healthy Koreans have been scarce compared to overseas studies, and most of them have focused on identifying associations with specific diseases^15,16^.

This study aimed to determine the serum and hair antioxidant trace mineral concentrations in healthy individuals and to investigate the differences in antioxidant trace mineral concentrations according to general characteristics. In addition, an association between serum or hair antioxidant trace minerals and the oxidative stress marker 8-OHdG was observed, which serves as a basis for future studies on the application of antioxidant trace minerals in predicting and preventing certain diseases associated with oxidative damage.

## Materials and methods

### Subjects

This study was approved by the Institutional Review Board of Bundang Jesaeng Hospital, Korea (project number: IMG14-03). Before the study, the subjects were informed of the purpose of the study, research procedures, expected benefits, possible risks, and precautions, and signed written informed consent was obtained. In addition, all procedures were performed in accordance with relevant guidelines.

**Figure 1:**
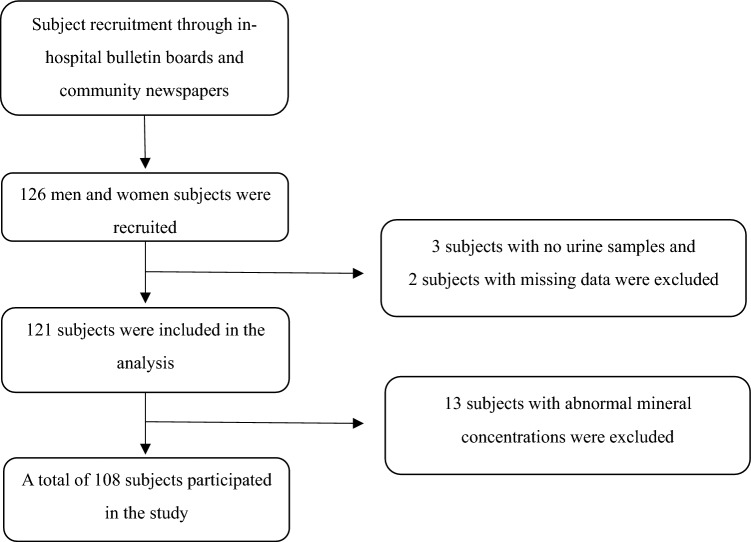
Flowchart of study subject selection.

Study participants were recruited through bulletin boards at Bundang Jesaeng Hospital and through local newspaper advertisements. Subjects were selected after screening, and 108 participants aged 19–69 years were finally included in the study. The exclusion criteria were as follows: (i) patients with results outside the reference range for general chemistry and blood tests; (ii) patients who were being treated or had been treated within the last three months for any of the following diseases: malignancy, diabetes, hypertension, thyroid disease, kidney disease, liver disease, renal disease, neurological disease, hematologic disease, and human immunodeficiency virus, hepatitis B virus, and hepatitis C virus infection; (iii) patients who had been treated for an infectious or inflammatory disease within the last month; and (iv) alcoholics or drug abusers. The overall flow of subject selection is shown in Fig. 1.

### General characteristics survey

The survey was administered to subjects by study staff. Subjects were randomly serialized, and they received no special dietary controls prior to sample collection. The characteristics included sex, age, body mass index (BMI), smoking, drinking, nutritional supplementation, exercise, dietary habits, and health status. Smokers are those who have smoked in the past and continue to smoke in the present, and drinkers are those who drink alcohol at least once a month, regardless of the type of alcohol. Exercisers exercised at least one hour per week, and the types of exercise ranged from walking, running, and jogging to table tennis, swimming, golf, cycling, and dancing. Supplement intake refers to the intake of supplements that do not contain antioxidant minerals (zinc, manganese, selenium, and chromium). Vitamins were the most common type of supplement, followed by probiotics and calcium. Health status was categorized as good or poor based on the subject's perception when filling out the lifestyle questionnaire. Regular eating is when you eat breakfast, lunch, and dinner at relatively the same time, while irregular eating is when you don't eat three meals a day or eat at irregular times.

### Serum collection and analysis of trace minerals

Venous blood (at least 4 mL) was collected, injected into a BD Vacuum Injector trace element serum container (Beckton Dickson, Franklin Lakes, USA), and placed at room temperature for 30 min. After 30 min, the blood was centrifuged at a maximum speed of 1300 × *g* for 10 min. The supernatant was separated, transferred to a cryo tube (screw type, 2 mL), and stored below − 80 °C until analysis. The analysis pre-processing process is as follows. Label the polypropylene tubes according to the order of the assay. Add 3.6 mL of diluent and dispense 200 μL each of sample, standard, and calibration materials. Add 200 μL of 1% HNO_3_, mix, and use as a sample for analysis. The analysis was measured using inductively coupled plasma mass spectrometry (ICP-MS; NexION2000B, Perkin-Elmer Corporation, Foster, CA, USA). The analyzer conditions are shown in Table 1.

**Table 1:** Analysis condition of serum trace minerals (ICP-MS NexION2000B, PerkinElmer).

Separation	Condition
RF Power	1600 W
Argon nebulizer gas flow	Approximate 0.9–1 LPM
Deterctor mode	Dual
Measutement uints	Cps
Scan mode	Peak hopping
Blank subtraction	After internal standard
Curve type	Simple Linear
Sample units	μg/dL (Zn), ng/mL (Mn, Se), μg/L (Cr)
Sweeps/reading	15 (Zn), 25 (Mn, Se, Cr)
Readings/replicate	1
Replicates	3
Dwell time	50 ms (Zn, Mn), 100 ms (Se, Cr)
Gas mode	KED (Zn), DRC Ammonia (Mn, Cr), Standard (Se)
RPQ	0.65 (Zn), 0.75 (Mn), 0.25 (Se, Cr)

### Hair collection and analysis of trace minerals

Scalp hair samples were cut from three locations from the occipital region of the head (3–5 cm; at least 150 mg). For subjects who had permed, dyed, or bleached their hair before sample collection, hair samples were collected at least two weeks after those treatments. Hair samples were washed in the following procedure. Label the outside of each specially designated 50 mL Corning Centrifuge Tube with the daily assigned lab number for each specimen to undergo the laboratory wash process. Remove entire hair specimen from sample envelope and place into the appropriately marked 50 mL tube. Add 25 mL or additional volume if necessary, of Triton X-I, to cover sample entirely. Secure cap on tube and place on vortexer for 5 s of vigorous vortexing. Decant solution carefully without specimen loss and repeat process for a total of four times for each specimen. Decant solution upon completion of final wash step for each tube. Add approximately 25 mL of reagent grade Acetone to each 50 mL tube. Secure cap on tube and place on vortexer for a period of 5 s of vigorous mixing. Upon completion decant solution carefully without specimen loss and allow to drain. Add approximately 30 mL of DI Water to each 50 mL tube, secure cap and place in one of the large available UltraSonic Baths. Once all specimens or the maximum number of specimens have been placed into the bath, adjust bath timer to 10 min and begin sonication. At the end of each 10 min wash, decant solution carefully without specimen loss and repeat entire process. Upon completion of the third repetition, decant solution carefully without specimen loss. Add approximately 25 mL of reagent grade Acetone to each 50 mL tube. Secure cap on tube and place on vortexer for a period of 5 s of vigorous mixing. Repeat process for a total of two times for each specimen. Upon completion decant solution carefully without specimen loss and allow to drain. Remove caps from tubes and place uncapped tubes into pre-heated drying oven (75 Centigrade +/− 5 degrees) for a period of 15 min. Remove all tubes from oven and transfer each hair specimen into the appropriately marked cutting cups for the standard cutting and weighing (80 mg) procedures. After that, each hair sample was finely cut into 1–2 mm sections using stainless steel scissors and mixed to ensure homogeneity. The cut hair was weighed to the nearest 0.001 g on an analytical-grade balance (Ohaus Explorer, Ohaus Corporation, Parsippany, NJ, USA) and then placed in a uniquely labeled, single-use, sterile polypropylene test tube. An aliquot of concentrated 70% trace metal-grade nitric acid (Fox Instrapure, Fox Scientific, Alvarado, TX, USA) was dispensed into each test tube. The tubes were then capped with sterile, two-position test tube caps, racked into a sample holder, and placed into a computer-controlled microwave digestion system (MARS 5, CEM Corporation, Matthews, NC, USA). After the microwave digestion procedure was complete, the samples were rehydrated with a diluent consisting of 18 MΩ de-ionized water and a solution of gold and a trace of HCl acid. The samples were then re-capped and mixed in a vortex mixer to ensure a uniform solution. From that point, the finished samples were placed into test tube racks to await analysis. Quantitative analysis was performed by Inductively Coupled Plasma Mass Spectrometry using the Elan 6100 and Elan 9000 analytical systems (Perkin Elmer, Akron, OH, USA).

### Urine collection and 8-OHdG assay

At least 20 mL of urine was collected from the subjects who provided informed consent. Before performing centrifugation, 5 ml of urine was transferred to a BD Vacutainer plain tube with a yellow cap (Becton Dickinson, Franklin Lakes, USA) because creatinine in the urine must be measured to correct for differences in 8-OHdG concentration due to urine dilution. And then, remaining urine was centrifuged at 2000–5000 × *g* for 10–15 min. Only the supernatant was separated in Cryo tube (screw type 2 mL) and stored below − 80 °C until analysis. The analysis was performed using spot urine samples, and urinary 8-OHdG levels were measured using an Epoch microplate spectrophotometer (BioTek, Santa Clara, CA, USA) and a DNA/RNA oxidative damage enzyme-linked immunosorbent assay kit (501130; Cayman Chemical, Ann Arbor, MI, USA) according to the manufacturer’s instructions. Each sample was assayed in duplicate, and the results were expressed as the ratio of 8-OHdG to creatinine. Creatinine was measured using an Epoch microplate spectrophotometer (BioTek) and a urinary creatinine colorimetric assay kit (500701, Cayman Chemical), according to the manufacturer’s instructions.

### Statistical analysis

Statistical analyses of all the collected data were performed using SAS version 9.4 (SAS Institute Inc., Cary, NC, USA). Continuous variables are expressed as mean ± standard deviation, and categorical variables are expressed as n and percentage (%) to compare the characteristics of the study subjects. As data on serum and hair trace mineral levels were characterized by non-Gaussian distribution, median and 25–75 percentile boundaries (interquartile range, IQR) were used as descriptive statistic. One-way analysis of variance was used to compare the mean values of three or more groups; a two-tailed *t*-test was used to compare the mean values of two specific groups; and differences between categorical variables were assessed for significance using the chi-square test. To determine the association between serum or hair antioxidant trace minerals and 8-OHdG levels, Pearson’s correlation analysis was performed after adjusting for sex, age, BMI, smoking, drinking, exercise, health status, eating status, and supplement use as potential confounding factors. Statistical significance was set at p < 0.05.

## Results

In this study, the average age of all subjects was 42.51 years and BMI was 23.12 kg/m^2^. There were significant differences in BMI (p < 0.001), smoking (p < 0.001), drinking (p < 0.001), and supplement use (p < 0.05) between sexes; however, there were no significant differences in age, exercise, health status, or eating habits (Table 2).

**Table 2:** General characteristics of subjects.

Characteristics	Total (n = 108)	Men (n = 46)	Women (n = 62)	p-value
Age (years)	42.51 ± 13.22	40.61 ± 11.48	43.92 ± 14.30	0.200
19−39	29.74 ± 5.28	30.45 ± 5.28	29.12 ± 5.30	
40−59	48.94 ± 5.49	48.73 ± 5.44	49.12 ± 5.64	
≥ 60	63.79 ± 2.67	63.00 ± 2.83	63.92 ± 2.75	
BMI (kg/m^2^)^†^	23.12 ± 3.05	24.66 ± 2.87	21.98 ± 2.67	0.0001***
< 23	20.70 ± 1.58	21.31 ± 1.69	20.59 ± 1.55	
≥ 23	25.29 ± 2.33	25.37 ± 2.56	25.13 ± 1.85	
Smoking				
Current smoker	12 (11.11)	12 (100)	0 (0.00)	0.0001***
Non-smoker	96 (88.89)	34 (35.42)	62 (64.58)	
Drinking				
Drinker	73 (67.59)	41 (56.16)	32 (43.84)	0.0001***
Non-drinker	35 (32.41)	5 (14.29)	30 (85.71)	
Exercise				
Yes	48 (44.44)	23 (47.92)	25 (52.08)	0.317
No	60 (55.56)	23 (38.33)	37 (61.67)	
Health status				
Good	98 (90.74)	41 (41.84)	57 (58.16)	0.780
Poor	10 (9.26)	5 (50)	5 (50)	
Eating habits				
Regular	84 (77.78)	36 (42.86)	48 (57.14)	0.327
Irregular	24 (22.23)	10 (41.67)	14 (58.33)	
Supplement use				
Yes	21 (19.44)	13 (61.90)	8 (38.10)	0.046*
No	87 (80.56)	33 (37.93)	54 (62.07)	

p-values were evaluated using Student’s *t*-test and the chi-square test.

Data are presented as mean ± standard deviation or n (%).

*p < 0.05, ***p < 0.001 statistically significant.

^†^Body mass index.

Serum zinc (p < 0.01) and selenium (p < 0.05) levels decreased with age. However, serum manganese and chromium levels were not significantly different among age groups. Exercisers had higher urinary 8-OHdG levels (p < 0.05) and lower serum zinc levels (p < 0.01) than non-exercisers (Table 3). As shown in Table 4, hair antioxidant trace mineral concentrations were not significantly different by age, but hair manganese was higher with better health status (p < 0.01). In addition, hair chromium levels were higher in men than in women (p < 0.05), in drinkers than in non-drinkers (p < 0.05), and in subjects with regular dietary habits (p < 0.05). In the correlation analysis between urinary 8-OHdG and minerals in serum and hair according to general characteristics, urinary 8-OHdG levels were negatively correlated with hair zinc in subjects aged > 60 years (p < 0.05) and those with poor health status (p < 0.05), and positively correlated with hair chromium in subjects with irregular dietary habits (p < 0.05, Table 5).

**Table 3:** Urinary 8-OHdG and serum antioxidant trace mineral concentrations by characteristics of the subjects.

Variables	Criteria	Urinary 8-OHdG (ng/mg creatinine)	Serum minerals			
			Zn (μg/L)	Mn (μg/L)	Se (μg/L)	Cr (μg/L)
Total subjects, n = 108		130.83 ± 98.32	1003.71 ± 243.22	1.45 ± 0.75	156.43 ± 24.02	0.34 ± 0.21
IQR		106.29 (81.46–151.62)	969.82 (827.80–1170.50)	1.38 (1.12–1.64)	154.64 (141.33–170.21)	0.29 (0.22–0.39)
Sex	Men, n = 46	122.91 ± 77.75	997.51 ± 281.80	1.49 ± 0.83	158.01 ± 28.49	0.34 ± 0.19
	IQR	104.14 (75.56–146.81)	942.10 (764.65–1219.45)	1.36 (0.83–1.70)	155.83 (136.22–180.47)	0.31 (0.20–0.41)
	Women, n = 62	136.71 ± 111.43	1008.31 ± 212.45	1.42 ± 0.69	155.25 ± 20.25	0.35 ± 0.23
	IQR	107.43 (82.98–152.44)	989.51 (845.43–1155.60)	1.38 (1.16–1.55)	154.40 (142.65–167.49)	0.28 (0.22–0.39)
p-value		0.450	0.829	0.649	0.577	0.902
Age, years	19–39, n = 47	121.84 ± 71.72	1073.73 ± 258.52	1.56 ± 0.61	163.43 ± 23.31	0.37 ± 0.24
	IQR	111.79 (86.36–142.20)	1020.77 (865.62–1255.13)	1.38 (1.22–1.69)	161.64 (148.22–171.63)	0.31 (0.22–0.43)
	40–59, n = 47	133.22 ± 124.75	980.23 ± 217.45	1.46 ± 0.87	152.98 ± 24.65	0.34 ± 0.21
	IQR	98.27 (64.51–163.80)	981.12 (826.40–1146.48)	1.46 (0.99–1.66)	150.99 (133.86–168.65)	0.31 (0.20–0.37)
	≥ 60, n = 14	153.04 ± 73.87	847.45 ± 192.60	1.01 ± 0.55	144.48 ± 17.39	0.27 ± 0.08
	IQR	137.17 (87.42–200.01)	787.08 (707.60–1027.89)	0.79 (0.58–1.37)	145.80 (128.82–159.13)	0.25 (0.21–0.35)
p-value		0.571	0.006**	0.050	0.013*	0.330
BMI, kg/m^2^	< 23, n = 51	131.99 ± 95.12	1031.99 ± 222.43	1.46 ± 0.72	159.02 ± 22.50	0.37 ± 0.26
	IQR	115.15 (91.82–145.31)	981.12 (850.37–1207.68)	1.38 (1.19–1.55)	158.10 (143.84–171.01)	0.27 (0.21–0.43)
	≥ 23, n = 57	129.80 ± 10.93	978.41 ± 259.77	1.44 ± 0.78	154.10 ± 25.27	0.32 ± 0.16
	IQR	99.96 (77.98–167.48)	957.95 (763.28–1152.00)	1.34 (0.89–1.69)	150.53 (136.21–168.64)	0.31 (0.23–0.37)
p-value		0.746	0.171	0.276	0.164	0.309
Smoking	Smoker, n = 12	156.25 ± 109.90	987.98 ± 262.56	1.40 ± 0.76	168.41 ± 29.22	0.33 ± 0.15
	IQR	128.78 (91.57–177.16)	945.72 (779.98–1220.22)	1.35 (0.77–1.64)	165.53 (147.17–193.70)	0.28 (0.21–0.41)
	Non-smoker, n = 96	127.66 ± 96.95	1005.68 ± 242.10	1.45 ± 0.75	154.93 ± 23.03	0.35 ± 0.22
	IQR	104.21 (78.87–148.22)	969.82 (834.60–1166.74)	1.38 (1.13–1.64)	153.96 (139.77–168.50)	0.29 (0.22–0.39)
p-value		0.345	0.813	0.798	0.067	0.753
Drinking	Drinker, n = 73	129.42 ± 110.48	1007.50 ± 261.18	1.47 ± 0.82	155.69 ± 24.07	0.34 ± 0.23
	IQR	103.85 (73.64–138.81)	958.52 (826.98–1208.72)	1.36 (1.08–1.57)	154.12 (140.28–169.29)	0.27 (0.21–0.38)
	Non-drinker, n = 35	133.78 ± 67.57	995.81 ± 204.01	1.40 ± 0.57	157.97 ± 24.19	0.36 ± 0.18
	IQR	115.15 (89.17–189.59)	1013.37 (826.40–1098.91)	1.40 (1.15–1.69)	159.31 (141.66–171.63)	0.32 (0.24–0.43)
p-value		0.801	0.817	0.619	0.646	0.632
Exercise	Yes, n = 48	154.22 ± 128.69	928.31 ± 215.97	1.37 ± 0.76	152.59 ± 27.40	0.30 ± 0.15
	IQR	114. 67 (88.42–188.96)	882.72 (759.50–1068.79)	1.26 (0.75–1.63)	149.85 (131.16–167.32)	0.25 (0.20–0.36)
	No, n = 60	112.13 ± 59.49	1064.03 ± 248.60	1.51 ± 0.73	159.50 ± 20.66	0.38 ± 0.25
	IQR	101.84 (71.72–138.95)	1043.14 (861.73–1240.16)	1.40 (1.21–1.67)	159.54 (146.46–170.89)	0.31 (0.23–0.43)
p-value		0.040*	0.004**	0.329	0.151	0.050
Health status	Good, n = 98	135.01 ± 100.23	999.30 ± 244.94	1.41 ± 0.71	155.63 ± 24.15	0.34 ± 0.21
	IQR	109.66 (83.70–150.00)	969.82(821.92–1162.06)	1.36 (1.09–1.64)	153.96 (138.94–170.02)	0.30 (0.22–0.39)
	Poor, n = 10	89.90 ± 67.87	1046.94 ± 233.29	1.80 ± 1.05	164.27 ± 22.30	0.39 ± 0.28
	IQR	70.29 (30.17–164.68)	980.28 (842.76–1213.22)	1.45 (1.31–2.05)	164.92 (153.06–179.60)	0.27 (0.21–0.55)
p-value		0.168	0.558	0.122	0.280	0.490
Eating habits	Regular, n = 84	127.42 ± 99.44	982.48 ± 247.03	1.41 ± 0.76	154.05 ± 24.26	0.33 ± 0.18
	IQR	103.61 (78.87–144.81)	957.80 (783.68–1154.48)	1.36 (1.02–1.57)	151.17 (136.21–169.89)	0.30 (0.22–0.39)
	Irregular, n = 24	142.80 ± 95.38	1078.02 ± 218.15	1.58 ± 0.68	164.73 ± 21.63	0.40 ± 0.31
	IQR	125.76 (87.06–177.16)	1077.82 (864.22–1220.22)	1.45 (1.24–1.75)	162.20 (154.25–170.74)	0.29 (0.21–0.45)
p-value		0.502	0.090	0.324	0.054	0.259
Supplement use	Yes, n = 21	128.59 ± 98.31	970.57 ± 230.08	1.56 ± 0.70	154.25 ± 27.74	0.32 ± 0.15
	IQR	101.33 (77.98–150.28)	981.12 (793.97–1086.84)	1.38 (1.24–1.77)	149.88 (132.11–171.38)	0.32 (0.23–0.37)
	No, n = 87	131.38 ± 98.89	1011.71 ± 246.90	1.42 ± 0.76	156.95 ± 23.19	0.35 ± 0.23
	IQR	107.52 (82.18–152.43)	958.52 (838.17–1184.86)	1.37 (1.06–1.57)	155.40 (142.98–170.30)	0.29 (0.21–0.41)
p-value		0.908	0.489	0.464	0.646	0.513

*p < 0.05, **p < 0.01 statistically significant.

p-values were calculated using one-way analysis of variance or Student’s *t*-test. Data are presented as mean ± standard deviation or median (interquartile range, IQR).

**Table 4:** Hair antioxidant trace mineral concentrations by characteristics of the subjects (n = 108).

Variables	Criteria	Hair minerals			
		Zn (μg/g)	Mn (μg/g)	Se (μg/g)	Cr (μg/g)
Total subjects, n = 108		181.67 ± 110.56	0.19 ± 0.28	0.60 ± 0.19	0.40 ± 0.18
IQR		160 (140–190)	0.12 (0.09–0.18)	0.60 (0.425–0.70)	0.40 (0.30–0.50)
Sex	Men, n = 46	189.35 ± 146.76	0.18 ± 0.35	0.63 ± 0.20	0.45 ± 0.22
	IQR	155 (140–180)	0.10 (0.08–0.17)	0.60 (0.50–0.80)	0.40 (0.30–0.50)
	Women, n = 62	175.97 ± 73.98	0.19 ± 0.21	0.58 ± 0.18	0.36 ± 0.13
	IQR	160 (140–200)	0.12 (0.09–0.18)	0.60 (0.40–0.70)	0.30 (0.30–0.43)
p-value		0.573	0.885	0.182	0.012*
Age, years	19–39, n = 47	191.49 ± 135.93	0.19 ± 0.36	0.58 ± 0.18	0.40 ± 0.22
	IQR	150 (140–190)	0.11 (0.08–0.15)	0.60 (0.40–0.70)	0.30 (0.30–0.40)
	40–59, n = 47	180.00 ± 95.46	0.17 ± 0.14	0.60 ± 0.19	0.41 ± 0.13
	IQR	170 (140–190)	0.13 (0.09–0.18)	0.60 (0.50–0.70)	0.40 (0.30–0.50)
	≥ 60, n = 14	154.29 ± 42.56	0.27 ± 0.33	0.67 ± 0.23	0.32 ± 0.16
	IQR	145 (130–185)	0.15 (0.07–0.31)	0.70 (0.40–0.90)	0.30 (0.20–0.33)
p-value		0.542	0.456	0.275	0.226
BMI, kg/m^2^	< 23, n = 51	180.39 ± 78.36	0.16 ± 0.16	0.56 ± 0.19	0.38 ± 0.13
	IQR	160 (140–200)	0.12 (0.08–0.16)	0.60 (0.40–0.70)	0.30 (0.30–0.50)
	≥ 23, n = 57	182.81 ± 133.80	0.21 ± 0.35	0.63 ± 0.19	0.42 ± 0.21
	IQR	150 (135–180)	0.12 (0.09–0.18)	0.60 (0.50–0.80)	0.40 (0.30–0.50)
p-value		0.908	0.327	0.066	0.316
Smoking	Smoker, n = 12	279.17 ± 266.00	0.33 ± 0.66	0.70 ± 0.22	0.56 ± 0.37
	IQR	175 (145–242.5)	0.12 (0.09–0.20)	0.75 (0.60–0.88)	0.40 (0.30–0.60)
	Non-smoker, n = 96	169.48 ± 64.99	0.17 ± 0.18	0.59 ± 0.18	0.38 ± 0.13
	IQR	155 (140–187.5)	0.12 (0.08–0.18)	0.60 (0.40–0.70)	0.40 (0.30–0.50)
p-value		0.182	0.414	0.051	0.120
Drinking	Drinker, n = 73	187.12 ± 122.89	0.18 ± 0.29	0.60 ± 0.19	0.42 ± 0.20
	IQR	160 (140–180)	0.11 (0.09–0.16)	0.60 (0.50–0.70)	0.40 (0.30–0.50)
	Non-drinker, n = 35	170.29 ± 79.32	0.21 ± 0.24	0.59 ± 0.19	0.35 ± 0.11
	IQR	150 (130–200)	0.13 (0.09–0.19)	0.60 (0.40–0.70)	0.30 (0.30–0.40)
p-value		0.393	0.563	0.829	0.031*
Exercise	Yes, n = 48	186.67 ± 122.50	0.19 ± 0.20	0.61 ± 0.21	0.38 ± 0.17
	IQR	170 (140–187.5)	0.13 (0.09–0.19)	0.60 (0.40–0.78)	0.35 (0.30–0.50)
	No, n = 60	177.67 ± 100.88	0.19 ± 0.33	0.59 ± 0.18	0.41 ± 0.19
	IQR	155 (140–190)	0.12 (0.08–0.17)	0.60 (0.50–0.70)	0.40 (0.30–0.50)
p-value		0.676	0.953	0.611	0.503
Health status	Good, n = 98	180.61 ± 114.49	0.20 ± 0.29	0.61 ± 0.19	0.39 ± 0.18
	IQR	155 (140–180)	0.12 (0.09–0.18)	0.60 (0.48–0.70)	0.40 (0.30–0.50)
	Poor, n = 10	192.00 ± 62.68	0.10 ± 0.04	0.53 ± 0.19	0.41 ± 0.12
	IQR	185 (157.5–230)	0.09 (0.08–0.13)	0.50 (0.40–0.63)	0.40 (0.30–0.53)
p-value		0.758	0.003**	0.220	0.801
Eating habits	Regular, n = 84	177.14 ± 88.27	0.19 ± 0.30	0.62 ± 0.19	0.41 ± 0.19
	IQR	160 (140–190)	0.12 (0.08–0.18)	0.60 (0.50–0.70)	0.40 (0.30–0.50)
	Irregular, n = 24	197.50 ± 168.56	0.17 ± 0.17	0.55 ± 0.19	0.35 ± 0.11
	IQR	155 (132.5–187.5)	0.12 (0.09–0.16)	0.60 (0.40–0.68)	0.30 (0.30–0.40)
p-value		0.574	0.625	0.112	0.039*
Supplement use	Yes, n = 21	188.57 ± 141.82	0.14 ± 0.12	0.57 ± 0.19	0.37 ± 0.09
	IQR	150 (140–175)	0.10 (0.07–0.16)	0.60 (0.40–0.65)	0.40 (0.30–0.40)
	No, n = 87	180.00 ± 102.55	0.20 ± 0.30	0.61 ± 0.19	0.40 ± 0.19
	IQR	160 (140–190)	0.12 (0.09–0.18)	0.60 (0.50–0.70)	0.40 (0.30–0.50)
p-value		0.796	0.140	0.443	0.206

*p < 0.05, **p < 0.01 statistically significant.

p-values were calculated using one-way analysis of variance or Student’s *t*-test. Data are presented as mean ± standard deviation or median (interquartile range, IQR).

**Table 5:** Pearson’s correlation coefficients between urinary 8-OHdG and antioxidant trace minerals by characteristics of the subjects.

Variables		Serum minerals				Hair minerals			
		Zn	Mn	Se	Cr	Zn	Mn	Se	Cr
Total subjects, n = 108		0.016 (0.876)	0.089 (0.381)	0.021 (0.834)	− 0.051 (0.614)	− 0.059 (0.564)	− 0.016 (0.872)	− 0.024 (0.814)	− 0.060 (0.553)
Sex	Men, n = 48	0.026 (0.877)	0.289 (0.079)	0.104 (0.536)	0.118 (0.479)	− 0.288 (0.079)	− 0.098 (0.559)	− 0.004 (0.980)	− 0.272 (0.098)
	Women, n = 64	0.026 (0.850)	− 0.011 (0.937)	− 0.042 (0.762)	− 0.135 (0.326)	0.120 (0.383)	0.013 (0.926)	− 0.029 (0.834)	0.055 (0.692)
Age, years	19–39, n = 47	− 0.019 (0.909)	− 0.069 (0.682)	− 0.112 (0.504)	− 0.095 (0.571)	− 0.038 (0.820)	− 0.044 (0.794)	0.013 (0.939)	0.137 (0.412)
	40–59, n = 47	− 0.113 (0.500)	0.049 (0.770)	− 0.045 (0.789)	− 0.115 (0.493)	− 0.134 (0.423)	− 0.050 (0.764)	− 0.033 (0.842)	− 0.153 (0.359)
	≥ 60, n = 14	0.798 (0.057)	0.768 (0.075)	0.641 (0.171)	− 0.629 (0.181)	** − **0.901 (0.014*)	− 0.576 (0.232)	− 0.591 (0.217)	0.642 (0.169)
BMI, kg/m^2^	< 23, n = 51	− 0.090 (0.571)	− 0.043 (0.788)	− 0.153 (0.333)	− 0.127 (0.422)	0.161 (0.310)	0.037 (0.816)	0.026 (0.869)	0.103 (0.516)
	≥ 23, n = 57	0.178 (0.225)	0.236 (0.106)	0.217 (0.138)	0.089 (0.545)	− 0.067 (0.650)	0.019 (0.900)	− 0.069 (0.641)	− 0.035 (0.813)
Smoking	Smoker, n = 12	− 0.476 (0.340)	− 0.466 (0.352)	− 0.483 (0.331)	0.088 (0.869)	− 0.581 (0.227)	0.176 (0.739)	− 0.479 (0.336)	− 0.122 (0.818)
	Non − smoker, n = 96	0.042 (0.694)	0.138 (0.199)	0.006 (0.952)	− 0.058 (0.593)	0.136 (0.208)	0.006 (0.957)	0.007 (0.952)	− 0.034 (0.754)
Drinking	Drinker, n = 73	0.099 (0.433)	0.062 (0.625)	0.044 (0.730)	− 0.073 (0.563)	− 0.136 (0.279)	− 0.026 (0.837)	− 0.086 (0.498)	− 0.090 (0.476)
	Non − drinker, n = 35	− 0.241 (0.216)	0.149 (0.450)	0.007 (0.970)	− 0.088 (0.658)	0.260 (0.182)	0.120 (0.542)	− 0.175 (0.374)	0.151 (0.444)
Exercise	Yes, n = 48	0.132 (0.418)	− 0.003 (0.985)	0.106 (0.515)	− 0.134 (0.411)	− 0.114 (0.484)	− 0.040 (0.807)	− 0.119 (0.466)	− 0.092 (0.574)
	No, n = 60	− 0.146 (0.355)	0.204 (0.195)	− 0.019 (0.905)	0.006 (0.972)	0.030 (0.849)	0.114 (0.471)	0.092 (0.564)	− 0.067 (0.673)
Health status	Good, n = 98	0.021 (0.846)	0.019 (0.862)	0.043 (0.690)	− 0.077 (0.469)	− 0.065 (0.545)	− 0.035 (0.743)	− 0.056 (0.601)	− 0.071 (0.509)
	Poor, n = 10	− 0.452 (0.701)	0.908 (0.275)	0.327 (0.788)	− 0.879 (0.317)	** − **0.997 (0.047*)	− 0.851 (0.351)	0.781 (0.429)	− 0.275 (0.822)
Eating habits	Regular, n = 84	0.065 (0.575)	0.112 (0.337)	0.120 (0.301)	− 0.109 (0.348)	0.001 (0.991)	− 0.031 (0.793)	− 0.060 (0.607)	− 0.172 (0.138)
	Irregular, n = 24	− 0.193 (0.475)	− 0.176 (0.513)	− 0.378 (0.149)	− 0.123 (0.649)	− 0.234 (0.382)	− 0.261 (0.329)	0.147 (0.588)	0.554 (0.026*)
Supplement use	Yes, n = 21	− 0.299 (0.320)	0.333 (0.267)	− 0.366 (0.218)	0.094 (0.759)	− 0.370 (0.213)	− 0.447 (0.126)	− 0.464 (0.110)	− 0.121 (0.694)
	No, n = 87	0.048 (0.673)	0.096 (0.400)	0.038 (0.740)	− 0.049 (0.671)	0.006 (0.957)	0.055 (0.628)	− 0.023 (0.843)	0.020 (0.861)

*p < 0.05, statistically significant.

R and p-values are presented after adjusting for sex, age, body mass index, smoking, drinking, exercise, health status, eating status, and supplement use. R-values greater than 0 indicate a positive correlation, whereas R-values smaller than 0 indicate a negative correlation.

*BMI* body mass index.

## Discussion

Considering the importance of antioxidant capacity in oxidative stress defense in the body, this study investigated blood and hair levels of potential antioxidant trace minerals and their associations with the oxidative stress marker 8-OHdG according to various health parameters. Different levels of serum or hair trace minerals were observed according to subjects’ characteristics such as age or exercise. We found significant associations of hair zinc and chromium with urinary 8-OHdG among older participants, those with poor health status, and those with an irregular diet.

The mean serum zinc concentration in this study was 1003.71 μg/L, which is within the range reported in published Korean studies (600–1900 μg/L)^17,18^. Hair zinc concentrations (mean of 181.67 μg/g) also ranged within the reference range of Medinex Korea^19^, which performed the hair test, and corresponded with values ranging from 150 to 210 μg/g in published Korean studies^19–21^. An international study also reported a range of 120–250 μg/g^22^. Zinc concentration may vary depending on factors such as age, BMI (obesity status), and residence, with younger people^23,24^, those who are not obese^19^, those who exercise^25^, and those who live in cities^26^ showing higher zinc concentrations. In this study, serum zinc concentration differed significantly with age. Serum zinc concentration also tended to decrease with increasing BMI; however, the difference was not significant. Despite not showing a significant difference, the concentration change with BMI followed the same trend as that reported in a previous study^27^. A sustained deficiency of zinc may lead to increased fat mass and decreased lean body mass. In obesity, zinc metabolism is prone to instability, with reduced absorption in the small intestine and increased excretion from the body^28,29^. Therefore, zinc concentration is of particular interest in the context of obesity. Additionally, serum zinc concentration was significantly different depending on the exercise status. Serum zinc concentrations in the exercise group were lower than those in the control group, which may have been due to higher 8-OHdG concentrations in the exercise group than in the control group. These findings suggest that serum zinc acts as an antioxidant against exercise-induced stress. Compared to previous studies on exercise, in one case, the control group had higher levels than the exercise group^30^, but in other cases, the exercise group had higher levels than the control group^31^. These differences may be due to interactions with other factors, such as age, sex, and diet, which may influence serum zinc concentration. Therefore, we suggest that further studies should be conducted to investigate changes in concentration with exercise after controlling for these factors. As shown in the results above, this study showed that serum zinc concentration was significantly different from the general characteristic factors, but hair zinc concentration was not.

In the present study, serum manganese concentration was 1.45 μg/L, in line with the range reported in previous studies (0.7–3.4 μg/L)^32,33^. Hair manganese concentration averaged 0.19 μg/g, which was slightly below the reference range of Medinex Korea^19^. Compared to the concentrations reported in domestic and international studies (average 0.15–0.4 μg/g), the result was within the range^19,20,34,35^. In this study, serum manganese concentration tended to decrease with age, although the difference was not significant. Hair manganese levels were significantly higher in those in good health than in those in poor health. Manganese is a particularly diet-sensitive nutrient, characterized by a decrease in the body owing to low food intake or when its bioavailability is reduced in conditions such as disease^36^. Manganese concentration may also be influenced by occupation and environment, with welding workers and those living near steel industrial parks having higher manganese concentrations than control subjects^32,37^. Excessive accumulation of manganese in the body has been reported to cause anemia and to negatively affect the nervous system^38^. Constant exposure to metals in the environment or due to occupation necessitates close attention to this factor.

The mean serum selenium concentration in this study was 156.43 μg/L, corresponding with the range reported in Korean studies (84–284 μg/L)^39,40^. Comparisons with other countries (average 67–136 μg/L), including China, Malaysia, Syria, Iran, and Sweden, indicated higher concentrations in Korea^41^. Differences in concentrations between countries may be attributed to diet and environment. Hair selenium in this study averaged 0.60 μg/g, within the reference range of Medinex Korea^19^. Serum selenium concentrations decreased with increasing age and were significantly different between the groups. The results of published studies on selenium concentrations with age vary. We found some studies that showed a positive correlation^42^, others that showed either a negative correlation^43^ or no association^44^. Different age ranges and characteristic dietary profiles, as well as different health conditions of the sample population, may have triggered these discrepancies.

The serum chromium concentration in this study was 0.34 μg/L, slightly lower than the range (0.37–3.96 μg/L) reported in domestic and international studies^45–47^. Hair chromium concentration averaged 0.40 μg/g, falling within the reference range of Medinex Korea^19^ and that of domestic and international studies (0.15–2.2 μg/g). Serum chromium concentration was not significantly different from the general characteristic factors; however, hair chromium concentration differed significantly according to sex (men > women), alcohol consumption (drinker > non-drinker), and eating habits (regular > irregular). Hexavalent chromium is toxic, whereas trivalent chromium has beneficial effects in humans. Drinking alcohol sensitizes the organs of the body, such as the liver, to hexavalent chromium toxicity^48^. In this study, 89% of the men and 52% of the women were drinkers. Sex differences in alcohol consumption may have influenced hair chromium concentration. However, there have been only few clinical and mechanistic studies on the relationship between alcohol consumption and hair chromium concentration^49^. Therefore, further studies are needed to understand the mechanisms of action and association between alcohol and chromium.

The mean urinary 8-OHdG level was 130.83 ng/mg creatinine. When comparing men and women, men had a mean concentration of 122.91 ng/mg creatinine, and women had a mean concentration of 136.71 ng/mg creatinine, with women having a slightly higher mean concentration than men, but there was no significant difference between the sexes. A review of published studies suggests that establishing a reference range for 8-OHdG concentrations may be difficult because it is sensitive to sex, age, body composition, smoking status, diet, ionizing radiation^50,51^, and occupational and environmental factors, resulting in a wide range of measured concentrations and difficulty in assessing the impact of only one factor^52^. In one clinical study, urinary 8-OHdG concentration increased with age^53^, whereas other studies showed no difference^54^. A comparison of urinary 8-OHdG concentration in smokers and non-smokers showed that smokers had 2.84 times higher concentration of urinary 8-OHdG than non-smokers among subjects with a BMI of ≤ 25^50^. Another study examining changes in 8-OHdG concentration by alcohol consumption and exercise did not show a significant correlation, but an association was observed between 8-OHdG concentration and nutritional supplement intake^55^. Additional studies are required to determine the sensitivity of this marker to environmental factors and nutritional status.

In the present study, 8-OHdG concentrations were significantly higher in exercisers than in non-exercisers. Although there were differences in the type and duration of exercise, previous studies have reported similar results^56,57^. We found no significant differences between 8-OHdG concentrations and any of the other general characteristic factors except exercise status. In the correlation analysis, 8-OHdG and hair zinc concentrations were negatively correlated in subjects aged > 60 years with poor health status, whereas 8-OHdG and hair chromium concentrations were positively correlated in subjects with irregular eating habits. However, no correlation was found between 8-OHdG concentration and serum antioxidant trace minerals. One possible explanation for this result is that the subjects in this study were relatively healthy adults who were influenced by defense systems other than antioxidant trace minerals^58^. However, it is possible that the study participants would have had different results if their concentrations of trace antioxidant minerals were below or above this range. Accordingly, our finding of a significant association between hair zinc and chromium levels and urinary 8-OHdG levels in more vulnerable subjects with older age, poorer health status, and irregular diet is significant. This was a cross-sectional study with limitations in the causal interpretation of these associations, and there is a need for future longitudinal studies to elucidate the relationship between these minerals and various oxidative markers.

We simultaneously determined antioxidant trace minerals in the serum and hair and compared the antioxidant trace mineral concentrations between groups within the general characteristics. Some results showed significant differences in concentrations based on general characteristics. In addition, we correlated serum and hair antioxidant trace mineral concentrations with oxidative stress markers and found a partially significant correlation. However, the limitations of this study include the fact that participants were recruited from a limited space in and around a university hospital; therefore, the sample size was small and not representative of the entire population. Second, only one oxidative stress marker, 8-OHdG, was used, and a variety of other oxidative stress markers were not included. Third, the study did not include an assessment of the subjects’ intake of macrominerals and antioxidant trace minerals. Fourth, because this study was conducted in normal subjects, a study of normal subjects with a wide range of measured concentrations and a comparative study of antioxidant trace mineral concentrations between normal and abnormal subjects is required. Despite these limitations, this study is significant for several reasons. First, this study compared antioxidant trace mineral concentrations between groups within general characteristic factors in healthy subjects, which may be used as a basis for future observational studies on antioxidant trace minerals according to general characteristics and may have applications in healthcare policy or clinical practice. Second, we observed correlations between serum or hair antioxidant trace minerals and 8-OHdG levels, with some results showing significant correlations. These results may be used as a basis for evaluating the applicability of antioxidant trace minerals to specific disease groups or population groups associated with oxidative stress, and if large epidemiological studies are conducted, this results may have generalizability.

## Conclusion

Serum zinc and selenium concentrations decreased with age; in particular, serum zinc concentrations were lower in exercisers than in non-exercisers. Hair manganese concentrations were higher in patients with good health status than in those with poor health status, and hair chromium concentration differed significantly according to sex (male > female), alcohol consumption (drinker > non-drinker), and eating habits (regular > irregular). Urinary 8-OHdG concentrations were higher in exercisers than in non-exercisers. In addition, in correlation analysis, urinary 8-OHdG showed a negative association with hair zinc concentration for the age of ≥ 60 years and poor health status, and a positive association with hair chromium in subjects with irregular diets. Future epidemiological studies with larger numbers of participants, particularly analyses including multiple markers of oxidative stress, are needed to confirm the applicability of these findings.
